# Exploring the Complex Landscape of Delayed Childbearing: Factors, History, and Long-Term Implications

**DOI:** 10.7759/cureus.46291

**Published:** 2023-09-30

**Authors:** Siddharth Zabak, Ashish Varma, Spandan Bansod, Meera R Pohane

**Affiliations:** 1 Pediatrics, Jawaharlal Nehru Medical College, Datta Meghe Institute of Higher Education and Research, Wardha, IND; 2 Department of Obstetrics and Gynecology, Smt. Radhikabai Meghe Memorial College of Nursing, Datta Meghe Institute of Higher Education & Research, Wardha, IND; 3 Medical Surgical Nursing, Shalinitai Meghe College of Nursing, Datta Meghe Institute of Higher Education and Research, Wardha, IND

**Keywords:** policy implications, work-life balance, reproductive choices, socioeconomic factors, parenthood trends, delayed childbearing

## Abstract

This review article delves into the intricate landscape of delayed childbearing, shedding light on the factors influencing individuals' decisions to postpone parenthood. In a world undergoing rapid social, economic, and technological transformations, the concept of when and why to become a parent has evolved significantly. We explore historical trends, societal norms, psychological dynamics, policy implications, and prospects surrounding delayed childbearing. This review underscores the diverse influences shaping this trend, from economic considerations and changing cultural perspectives to advancements in reproductive technologies and the complexities of work-life balance. By examining the emotional dimensions and long-term consequences, we comprehensively understand the implications for individuals, families, and societies. As we conclude, we emphasize the importance of addressing challenges and embracing opportunities to create a supportive environment for those navigating the complex decisions tied to delayed childbearing.

## Introduction and background

Delayed childbearing, a prominent trend in recent decades, reflects substantial shifts in societal norms, economic structures, and individual aspirations. Traditionally, childbearing occurred within a relatively narrow age range, driven primarily by cultural norms and biological considerations. However, the 21st century has witnessed a noticeable increase in the average age at which individuals embark on parenthood [1]. This phenomenon, commonly called delayed childbearing, has instigated a growing body of research to comprehend the multifaceted factors contributing to this trend [2,3].

Delayed childbearing, as a concept, emerged as a response to the evolving landscape of women's lives and aspirations. It refers to the trend of individuals, particularly women, choosing to postpone childbearing until later in life, often beyond their 20s and into their 30s or even 40s. This phenomenon challenges traditional notions of when parenthood should occur and is characterized by various factors influencing individuals' decisions [2]. As the world modernizes and societies become more diverse and interconnected, delayed childbearing is no longer limited to specific demographics or regions. It is a global trend that reflects the complex interplay between personal choices, cultural values, technological advancements, and economic considerations [3].

The decision to delay childbearing is a complex interplay of personal choices, sociocultural influences, technological advancements, and health-related considerations. As this trend progresses, it becomes imperative to delve deeper into the underlying factors driving this societal shift. Researchers, policymakers, and healthcare professionals can gain valuable insights into the broader implications for individuals, families, and societies by comprehensively examining the reasons behind delayed childbearing [4-6].

Understanding the factors influencing delayed childbearing is not solely an academic pursuit; it holds significant societal ramifications. These consequences extend from the dynamics of the labor market to the structure of family units, from formulating public policies to providing reproductive healthcare services. Furthermore, insights into delayed childbearing illuminate the evolving nature of gender roles, family dynamics, and individual aspirations, contributing to a more nuanced understanding of contemporary social dynamics [7].

This review article explores factors contributing to the growing phenomenon of delayed childbearing. In doing so, it will structure the discussion into distinct sections: a concise background, an elucidation of delayed childbearing, its global distribution, factors influencing delayed childbearing, long-term consequences, the novelty of this review, the rationale behind conducting this review, and, finally, the aims and objectives of this study. Notably, we will clarify the primary and secondary objectives in the closing part of this introduction to provide a more focused outline for the reader.

By scrutinizing these factors from multiple perspectives, encompassing socioeconomic, cultural, technological, psychological, and policy dimensions, this article offers a comprehensive overview of the intricate web of influences shaping individuals' decisions regarding when to start a family. Our review encompasses a wide array of factors contributing to delayed childbearing, including the surge in women's educational and career aspirations, societal expectations surrounding motherhood, advancements in assisted reproductive technologies, evolving relationship dynamics, and the multifaceted health and medical considerations that influence fertility and pregnancy outcomes in later life. We aim to foster a deeper understanding of the motivations and challenges associated with delayed childbearing through an in-depth analysis of these factors. Additionally, we intend to highlight the policy implications stemming from this trend and suggest directions for future research to enrich our comprehension of this complex phenomenon further.

## Review

Historical context

Exploring Historical Trends in Childbearing Age

Throughout history, childbearing age has been influenced by a combination of biological, cultural, and socioeconomic factors. In agrarian societies, where survival and productivity were closely tied to reproduction, individuals tended to marry and bear children at a younger age. However, a gradual childbearing age shift began as industrialization and urbanization reshaped societies [8].

The late 19th and early 20th centuries witnessed a transition as access to education and employment opportunities for women expanded. This shift, coupled with improvements in healthcare and contraception, contributed to a shift in societal norms surrounding childbearing. Women began postponing childbirth to pursue education, careers, and personal aspirations [9,10].

Societal Norms and Expectations in Different Eras

Societal norms and expectations regarding childbearing have evolved across eras and cultures. In traditional societies, early marriage and parenthood were often emphasized, as they were essential for economic stability and the continuation of family lineage. These cultural expectations reinforced the notion that childbearing was a central role for women [11,12].

However, in the latter half of the 20th century, the feminist movement and changing perceptions of gender roles began challenging these norms. Women's increased participation in the workforce and the desire for greater autonomy shifted the balance between family and personal ambitions. These changes in societal values gradually opened the door to the concept of delayed childbearing [13-15].

Factors influencing delayed childbearing

Economic stability and career aspirations: In the wake of evolving societal dynamics, the interplay between economic stability and career aspirations has emerged as a fundamental driver of delayed childbearing [14]. The increasing participation of women in higher education and the workforce has given rise to a paradigm shift in personal goals. With career aspirations becoming paramount, individuals postpone parenthood to establish a firm financial security foundation and attain their professional ambitions [15]. This strategic delay allows individuals to amass the resources necessary to provide for their families comfortably while avoiding the potential conflict between child-rearing demands and career advancement. The desire for economic stability intertwines with the pursuit of personal accomplishments, presenting a compelling incentive for deferring the initiation of parenthood [16].

Education and opportunities for women: The expanding landscape of educational opportunities for women has catalyzed a transformative narrative in the context of delayed childbearing. With increased access to higher education and a plethora of professional career pathways, women are empowered to make informed choices about the timing of parenthood. Higher levels of educational attainment equip women with the skills, knowledge, and self-confidence necessary to explore their academic and career potential [16] fully. Consequently, the decision to delay childbearing becomes calculated, enabling women to harness their educational achievements and chart well-informed trajectories for their professional growth. This educational empowerment encourages women to defer parenthood until they can optimally balance their aspirations and parenting responsibilities [17].

Impact of financial considerations: The decision to postpone childbearing is intrinsically entwined with financial considerations, significantly impacting reproductive choices. The escalating costs associated with raising children, from prenatal care to education, underscore the importance of financial preparedness. As the financial burden of parenthood becomes more pronounced, individuals often choose to delay having children until they feel sufficiently equipped to provide a stable and nurturing environment [17]. The imperative of a stable income and adequate savings in the face of these mounting expenses is a primary motivation to postpone parenthood. This pragmatic approach aligns with the broader societal trends of establishing a solid financial foundation before embarking on the transformative journey of raising a family [18].

Changing cultural perspectives on motherhood: Cultural perspectives on motherhood have evolved throughout history, reflecting the broader shifts in societal values and gender roles. Traditionally, motherhood was regarded as a central and defining aspect of a woman's identity, often overshadowing other potential roles or aspirations. However, contemporary viewpoints recognize that women's identities are multifaceted and extend beyond their roles as mothers. This paradigm shift acknowledges that women possess diverse talents, ambitions, and passions beyond parenting. This transformation in cultural perspectives empowers women to consider personal goals, career aspirations, and individual experiences before embarking on the journey of parenthood. Recognising the complexity of women's identities allows for greater agency in deciding when and if to become mothers [19].

Influence of family values and societal pressures: Family values and societal pressures significantly influence individuals' decisions regarding childbearing. In cultures where early marriage and parenthood are traditionally prioritized, there can be a considerable clash between these norms and an individual's aspirations. This tension between familial or societal expectations and an individual's desires can lead to decisions to postpone parenthood. Individuals may feel torn between fulfilling the expectations of their families and communities and pursuing personal goals such as education, career advancement, or personal growth. Negotiating this balance can be challenging, as individuals strive to honor their cultural heritage while embracing the evolving possibilities of delayed childbearing [20].

Role of media and pop culture: Media and pop culture have played a transformative role in shaping societal perceptions and norms, including childbearing. The portrayal of successful women in various roles and pursuits beyond motherhood has reshaped traditional ideas of what constitutes a fulfilling life. Media representations of women who prioritize careers, travel, creative endeavors, and personal growth have highlighted the potential richness of life experiences that can be pursued before parenthood. Moreover, the visibility of celebrities and public figures who delay childbearing for reasons such as education, career development, or personal fulfillment has normalized the concept. These public narratives help challenge the notion that parenthood should be women’s primary and immediate goal, encouraging others to explore their potential before embracing the responsibilities of raising a family [21].

Technological advancements

Assisted reproductive technologies (ARTs): The landscape of ARTs has undergone transformative changes driven by innovations such as in vitro fertilization (IVF) and egg freezing. These advancements have emerged as powerful tools, granting individuals unprecedented control over their reproductive timelines. ARTs can sometimes inadvertently contribute to a delay in childbearing. For instance, IVF combines eggs and sperm outside the body, enabling fertilization before transferring embryos to the uterus. While IVF has revolutionized family planning by helping couples overcome fertility challenges, it can also assure individuals that they can conceive at a later age. This confidence may lead some people to delay starting a family, believing they can always turn to IVF when ready [22].

Similarly, egg freezing, a pivotal aspect of ARTs, empowers women by allowing them to preserve their eggs at a younger age when fertility potential is higher. These preserved eggs can be thawed, fertilized, and implanted, offering women a means to extend their childbearing years and pursue life goals before embracing parenthood [22]. However, this option can inadvertently encourage women to postpone childbirth while focusing on career advancement or other life pursuits, assuming their fertility remains preserved. In practical terms, consider a scenario where a woman in her late twenties decides to freeze her eggs to ensure her fertility. With this safety net in place, she may delay starting a family and instead invest more time in her career or personal development, knowing she can rely on her preserved eggs later. While this is a valuable choice, it exemplifies how ARTs, like egg freezing, can influence the timing of childbearing decisions. Therefore, it's essential to recognize that while ARTs provide valuable reproductive options and flexibility, they can also contribute to delayed childbearing by offering individuals a sense of security and control over their reproductive choices [23].

Fertility preservation techniques: Fertility preservation techniques, exemplified by oocyte cryopreservation or egg freezing, have ushered in a new era of reproductive possibilities. This technique involves extracting a woman's eggs, freezing them, and storing them until a later time. This innovation is particularly significant for individuals facing medical treatments that may compromise their fertility, such as chemotherapy, or those navigating career trajectories that necessitate delaying childbearing. Egg freezing provides a biological safeguard and an empowerment tool, allowing women to align their reproductive decisions with their life circumstances. By offering the potential to utilize preserved eggs at a future point, fertility preservation techniques offer an avenue to mitigate the impact of time on fertility and support more informed family planning choices [23].

Impact on extending the biological clock: The synergy of technological advancements and medical interventions has notably extended the biological clock for women. While it is well-established that fertility diminishes with age due to a decline in egg quantity and quality, the advent of ARTs has offered a measure of counterbalance. By leveraging the advances in reproductive science, these technologies have allowed individuals to conceive beyond what nature's timeline might otherwise allow. This extension of the biological clock is flexible, as fertility naturally declines with age. However, technological strides have created an avenue for individuals to pursue parenthood later in life, blurring the lines of traditional age-related constraints and offering new horizons for those who wish to optimize their reproductive potential [24].

Relationship dynamics

Delayed marriages and implications for childbearing: In the landscape of modern relationships, the age at which individuals decide to enter into marriage has significantly shifted, subsequently influencing the timeline of parenthood. As societal norms adapt to changing opportunities and priorities, many individuals emphasize personal growth, educational pursuits, and the establishment of their careers before considering marriage. This trend, known as delayed marriage, inherently impacts the decision to start a family. As couples delay marriage to achieve personal and professional milestones, the subsequent delay in childbearing follows suit. This phenomenon holds implications for family planning, as the biological window for childbearing narrows with advancing age [25].

Evolution of partnership dynamics: The landscape of partnership dynamics has transformed remarkably, reflecting the diverse ways individuals form relationships and build families. Traditional models of marriage have expanded to include cohabitation, non-traditional partnerships, and diverse family structures. These shifts in partnership dynamics are pivotal in influencing decisions surrounding childbearing. In non-traditional relationships, considerations about parenthood often intertwine with personal aspirations, financial stability, and relationship dynamics. The evolution of partnership models underscores the need for tailored approaches to family planning and highlights the intricacies that individuals and couples navigate in deciding when to become parents [26,27].

Co-parenting in non-traditional family structures: Co-parenting has gained traction within non-traditional partnership arrangements, reshaping family planning strategies. Individuals in relationships that do not conform to the conventional norms of marriage may opt for co-parenting arrangements, where partners may not share a romantic relationship but collaborate to raise children. In these unique family structures, delayed childbearing takes on a distinct dimension. The flexibility of co-parenting arrangements allows individuals to carefully consider when and how they choose to embark on parenthood, aligning the decision with their personal and relational circumstances. This can lead to a deliberate delay in childbearing as individuals navigate the complexities of co-parenting dynamics [28,29].

Health and medical considerations

Age-related fertility decline and associated risks: Biological factors profoundly affect the decision to delay childbearing. As women age, their reproductive capacity diminishes due to decreased quantity and quality of eggs. This decline in fertility is a natural consequence of the aging process, and women who opt to delay childbearing may face challenges when trying to conceive. The biological clock ticks faster as women move into their late 30s and 40s, making it more difficult to achieve pregnancy and increasing the likelihood of infertility. Moreover, the risks associated with pregnancy complications rise significantly with advancing maternal age. Older mothers are at an elevated risk of miscarriages, ectopic pregnancies, and conditions like placenta previa. Additionally, the risk of chromosomal abnormalities in the developing fetus, such as Down syndrome, substantially increases as maternal age rises [30,31].

Maternal and fetal health in older pregnancies: Delayed childbearing brings about medical considerations for mothers and their unborn children. Advanced maternal age, often associated with delayed childbearing, can have a pronounced impact on maternal health. Pregnant women in their late 30s and beyond are at an increased risk of developing gestational diabetes, which can lead to complications for both mother and child. Preeclampsia, characterized by high blood pressure and organ damage, is more common among older pregnant women and poses significant health risks. Notably, the mother’s age also plays a role in fetal health. Older maternal age is correlated with a higher incidence of chromosomal abnormalities and congenital disabilities in newborns. Maternal and fetal health complexities in older pregnancies highlight the importance of informed decision-making and comprehensive prenatal care [32].

Advantages and challenges of medical interventions: Advancements in medical technology have provided hope for those who choose delayed childbearing. Prenatal screening and genetic testing offer valuable insights into the health of the developing fetus, helping parents make informed decisions. These interventions can identify chromosomal abnormalities and genetic disorders early in pregnancy, allowing individuals to prepare for potential challenges or consider alternatives if necessary. However, the advantages of medical interventions come hand in hand with ethical, emotional, and financial dilemmas. For instance, the decision to undergo prenatal testing may raise ethical concerns about selective abortion based on the results. Couples considering delayed childbearing must navigate the emotional complexities of these choices, and the costs associated with these medical interventions can pose a financial burden. Striking a balance between the benefits and challenges of medical interventions is a crucial aspect of the decision-making process for those contemplating delayed childbearing [33,34].

Psychological and emotional factors

Ambivalence and Decision-Making

Mixed feelings about parenthood: Delayed childbearing often ushers in a complex and profound ambivalence among individuals. While the aspiration to embrace parenthood is prevalent, this sentiment can be intertwined with a tapestry of conflicting emotions. Becoming a parent is laden with uncertainties and complexities, which can evoke various feelings. On the one hand, the desire to experience the joys of nurturing a family is present; on the other hand, concerns about how parenting might disrupt existing personal goals and lifestyles emerge. This emotional ambivalence introduces layers of intricacy into the decision-making process. Individuals grapple with weighing the fulfillment of their ambitions against the profound commitment and responsibilities of raising children. This ambivalence often postpones childbearing until a more opportune and balanced time in their lives [35].

Balancing personal goals with parenting desires: As individuals navigate the trajectory of delayed childbearing, the dynamic equilibrium between personal aspirations and the desire for parenthood takes center stage. Pursuing higher education, career advancement, and exploring the world through travel are among the diverse personal goals individuals seek to accomplish before embarking on parenthood. However, these ambitions can sometimes be at odds with the pull of parenthood. The endeavor to strike a harmonious balance between these seemingly divergent paths becomes an intricate negotiation. The decision of when to transition into parenthood requires carefully considering how individual aspirations coexist with the commitment to raising a family. This intricate negotiation exemplifies the delicate interplay between embracing one's individuality and fulfilling the role of a parent, ultimately shaping the timeline of delayed childbearing [36].

Psychological Well-Being

Impact of societal expectations on mental health: Societal expectations significantly influence individuals' mental well-being regarding delayed childbearing. Prevailing norms and attitudes regarding the “ideal” age for parenthood can create pervasive pressure and anxiety for those who postpone having children. The fear of being judged by peers, family, or society and the perception of deviating from established norms can lead to feelings of inadequacy and self-doubt. This phenomenon can contribute to adverse mental health outcomes, including heightened stress levels, lowered self-esteem, and increased vulnerability to conditions like anxiety and depression. Recognizing and addressing these societal pressures is vital in fostering positive mental health for individuals opting for delayed childbearing. Challenging the stigma surrounding unconventional timelines and promoting an inclusive dialogue can alleviate the psychological toll of societal expectations [37].

Coping strategies for emotional challenges: Opting for delayed childbearing can introduce individuals to a unique set of emotional challenges. The pressure to conform to societal norms while simultaneously striving to fulfill personal goals can create an internal conflict that affects emotional well-being. Individuals often adopt coping strategies that provide emotional support and resilience to navigate these complexities. Seeking solace in close relationships with friends and family, sharing concerns, and seeking advice can provide comfort. Additionally, engaging with mental health professionals who specialize in addressing the emotional intricacies of life decisions can offer valuable tools and strategies to manage emotional stressors effectively. These coping mechanisms are central to empowering individuals to navigate the emotional terrain of delayed childbearing while maintaining their mental equilibrium [38].

Parenting self-efficacy and identity: Delayed childbearing can significantly impact how individuals perceive themselves as potential parents. With the accumulation of life experiences, skills, and self-confidence, individuals often develop a heightened sense of preparedness to embrace the challenges and responsibilities of parenthood. The journey toward delayed parenthood, marked by personal growth and self-discovery, can instill a mature and self-assured approach to parenting. As individuals chart their path to parenthood, their parenting identity evolves in distinctive ways. The confidence gained through life experiences and the capacity to navigate complex life decisions can foster a sense of competency and self-efficacy in anticipating the responsibilities of raising a family. This more informed and intentional perspective can lead to enriched parenting experiences, enabling individuals to approach parenthood with a deeper understanding of themselves and their caregiver roles [39].

Policy implications

Work-Life Balance Policies

Parental leave and flexible work arrangements: Work-life balance policies, encompassing parental and flexible work arrangements, emerge as pivotal components in facilitating delayed childbearing for individuals seeking to balance their careers with family aspirations. Paid parental leave is a cornerstone of these policies, offering new parents the crucial opportunity to devote time to caring for their newborns without jeopardizing their professional trajectories. This form of leave fosters stronger parent-child bonds and acknowledges the significance of family life within the context of a professional career [40].

Flexible work arrangements constitute an additional facet of these policies that have gained prominence. These arrangements, which encompass remote work options, adjusted schedules, and part-time positions, present an adaptable approach that caters to the evolving needs of working parents. By accommodating the challenges of juggling family responsibilities and career demands, flexible work arrangements pave the way for individuals to transition into parenthood more easily [41].

Supportive Workplace Environments

A crucial facet of enabling delayed childbearing lies in creating supportive workplace environments that recognize the importance of family life. Organizations that invest in their employees' well-being and family needs exhibit a forward-thinking approach to modern workforce dynamics. By providing amenities like on-site childcare facilities and dedicated lactation rooms, employers foster an environment where the practical needs of working parents are met, alleviating the pressures associated with raising a family while managing professional responsibilities [42].

Furthermore, workplaces that offer parenting education resources contribute to employees' holistic well-being. Employers bolster the confidence and capabilities of individuals navigating the complexities of parenthood by facilitating access to guidance on parenting skills, child development, and work-life integration [43].

Influence on Career Trajectories

The profound impact of work-life balance policies on career trajectories cannot be overstated, particularly in the context of delayed childbearing. For women, in particular, these policies play a vital role in counteracting the “motherhood penalty,” a phenomenon where career advancement is hampered by the perception of diminished commitment due to parenting responsibilities. By implementing supportive policies that accommodate parenting leaves, flexible arrangements, and opportunities for continued professional development, organizations create an environment where individuals, regardless of gender, can pursue their career goals while fulfilling their aspirations for parenthood [44].

Access to reproductive healthcare

Availability of Fertility Treatments and Information

Ensuring widespread access to comprehensive reproductive healthcare services is a cornerstone of supporting individuals who opt to delay childbearing. This entails providing a spectrum of fertility treatments and counseling that cater to the specific needs and aspirations of those considering delayed parenthood. In a rapidly advancing field, individuals contemplating the postponement of parenthood can avail themselves of various options, including fertility preservation techniques and medical interventions. Fertility preservation, such as egg freezing, empowers individuals to safeguard their reproductive potential until they are ready to start a family. Access to accurate and informative resources about reproductive health also equips individuals with the knowledge necessary to make informed decisions aligned with their life goals [45].

Ethical Considerations in Assisted Reproduction

The rapid evolution of ARTs has introduced a host of ethical considerations that policymakers must grapple with. Innovations like egg freezing, surrogacy, and genetic testing have redefined family planning possibilities and raised complex ethical questions. Balancing the principles of individual autonomy, reproductive rights, and societal well-being is paramount. Policymakers are tasked with creating regulations that safeguard the rights of all parties involved while navigating issues related to consent, potential exploitation, and the well-being of both donors and recipients. Addressing these ethical concerns is crucial for ensuring these technologies' responsible and equitable use [46].

Health Insurance Coverage for Reproductive Services

The role of health insurance in shaping family planning decisions must be considered. Comprehensive health insurance coverage for reproductive services, particularly fertility treatments, enables individuals to make informed choices about delayed childbearing. With the high costs associated with fertility treatments and ARTs, insurance coverage mitigates financial barriers that might otherwise deter individuals from pursuing their desired timelines for parenthood. By providing access to these services, insurance coverage ensures that individuals have the necessary resources to navigate the complexities of fertility preservation and reproductive interventions, offering them a range of possibilities regardless of their socioeconomic status. This coverage empowers individuals to take control of their reproductive journey and aligns with the broader goal of promoting equitable access to healthcare services [47].

Family support and social infrastructure

Importance of familial and community networks: Familial and community support networks are pivotal pillars in facilitating the journey of delayed childbearing. In an increasingly diverse and dynamic society, these networks provide invaluable emotional, practical, and childcare assistance to individuals and couples navigating the complexities of parenthood at an older age. The presence of supportive family members, friends, and neighbors can mitigate the challenges that come with delayed childbearing. This support extends beyond mere assistance, encompassing a sense of belonging and shared experiences that foster resilience and mental well-being. Familial solid and community networks offer guidance, share wisdom, and provide a safety net that enables individuals to approach parenting with greater confidence, even in the face of potential hurdles [48].

Childcare facilities and support systems: Providing well-developed childcare facilities and comprehensive support systems is fundamental for individuals who opt for delayed childbearing. As more individuals choose to balance their family aspirations with career pursuits and personal ambitions, the availability of accessible and affordable childcare options becomes paramount. These facilities offer children a safe and nurturing environment and allow parents to engage in other pursuits, including education and professional growth. Access to reliable childcare helps alleviate the strain on parents, allowing them to maintain a work-life balance conducive to their personal and familial well-being. These support systems empower individuals to actively participate in parental roles while nurturing their aspirations and goals [49].

Government policies to promote family planning: Government policies aimed at promoting family planning and supporting the concept of delayed childbearing wield significant influence over the decisions of individuals and couples. Policies designed to incentivize delayed childbearing can take various forms, such as tax benefits, subsidies for fertility treatments, and comprehensive healthcare coverage. By acknowledging and supporting the diverse reproductive timelines of individuals, governments can contribute to creating an environment where personal and family aspirations align harmoniously. Public awareness campaigns about reproductive health and family planning options further empower individuals with information, allowing them to make informed choices about when and how to embark on parenthood. These policies reflect the evolving needs of modern society and lay the foundation for a future where delayed childbearing is embraced as a valid and respected choice [50].

Future directions and research gaps

Identifying areas for further research: As we explore the intricate complexities inherent to delayed childbearing, it becomes evident that several domains demand further scholarly inquiry and investigation. These uncharted research territories present opportunities to unveil hidden facets of this phenomenon, enriching our understanding and shaping informed approaches [51].

Cultural and regional variations: Exploring cultural norms, values, and regional disparities concerning delayed childbearing has the potential to unveil a global tapestry of influences. As cultures vary greatly in their expectations, beliefs, and societal structures, understanding how these factors intersect with the decision to delay parenthood can illuminate the worldwide scope of this trend. This examination may reveal the intricate ways in which local and global forces interplay, leading individuals to navigate the choice of parenthood within the context of their unique cultural landscapes [52].

Impact on fathers: While the focus of this review primarily centers on maternal aspects of delayed childbearing, an equally important avenue for investigation lies in understanding the experiences, decisions, and well-being of fathers. Exploring how delayed parenthood affects fathers can shed light on their evolving roles and responsibilities within the family unit. This inquiry may uncover how fathers navigate their aspirations, career trajectories, and personal development in conjunction with the decision to delay childbearing, contributing to a more holistic understanding of familial dynamics [53].

Intersectionality: The rich tapestry of human experiences is woven with the threads of various identities, including race, ethnicity, socioeconomic status, and sexual orientation. Investigating the intersections of these identities with delayed childbearing can unravel unique challenges and opportunities faced by diverse populations. The emerging disparities and commonalities can provide insights into how societal norms, expectations, and access to resources shape decisions to postpone parenthood among individuals with varying backgrounds. This exploration can deepen our comprehension of how systemic factors intersect with personal choices, fostering a more inclusive and nuanced understanding of delayed childbearing [54].

Long-term consequences of delayed childbearing

Health outcomes for parents and children: Understanding the health outcomes of parents who opt for delayed childbearing offers valuable insights into this trend's physical and emotional dimensions. As individuals postpone parenthood, their age at conception rises, which can influence maternal and paternal health. Research in this area explores the potential links between advanced parental age and health outcomes. It investigates the incidence of pregnancy-related complications, such as gestational diabetes, hypertension, and cesarean deliveries among older mothers. Additionally, it examines the psychological well-being of older parents, considering factors such as stress, emotional resilience, and the experience of raising children later in life. Equally significant is the examination of long-term effects on the offspring. Studies delve into the potential associations between delayed childbearing and children's health, developmental milestones, and psychological well-being, offering valuable insights into the holistic implications of this trend for both generations [55].

Social and economic implications: Delayed childbearing transcends the individual and family realms, encompassing broader social and economic dimensions. Investigating the social and economic implications provides a comprehensive view of how this trend interconnects with larger societal structures. For instance, the impact on intergenerational dynamics considers how delayed parenthood might affect relationships between parents and their adult children. Exploring the caregiving responsibilities that older parents might face as they age further illuminates family dynamics. From an economic standpoint, delayed childbearing can influence workforce participation and retirement patterns. Individuals who become parents later might intersect with their peak career years, potentially impacting productivity and retirement planning. By examining these implications, we gain insights into the potential ripple effects of delayed childbearing on various facets of society, from family structures to labor markets and social support systems [56].

Anticipating societal shifts and evolving trends

Technological advances and reproductive choices: The relentless progression of reproductive technologies stands to redefine the contours of delayed childbearing. As these technologies continue to evolve, they can revolutionize family planning options. With advanced fertility preservation techniques, individuals can extend their biological windows, fostering greater autonomy over when and how they choose to embark on parenthood. As these technologies become more accessible and refined, they will likely play a pivotal role in reshaping the landscape of delayed childbearing, offering new avenues, and expanding the range of reproductive choices available [57].

Policy adaptation: The dynamic interplay between delayed childbearing and policy frameworks is critical. As societal shifts transform family dynamics, policies that accommodate these changes must adapt. Continuous monitoring and evolution of policies to support delayed childbearing are paramount. This includes offering comprehensive reproductive healthcare and implementing work-life balance initiatives that facilitate the dual roles of career and parenthood. The responsiveness of policies to the evolving needs of individuals and families will be instrumental in promoting overall family well-being and societal cohesion [58].

Changing definitions of parenthood: Parenthood is undergoing a profound transformation. Delayed childbearing intersects with this evolution, offering fresh perspectives on traditional notions of family and parenting roles. As individuals prioritize personal goals and aspirations, they reshape the narrative around parenthood, highlighting the diverse pathways leading to family formation. Future research can explore how delayed childbearing contributes to this evolving definition of parenthood, embracing a spectrum of choices and fostering a more inclusive understanding of familial relationships [59].

## Conclusions

In conclusion, this comprehensive review has shed light on the intricate web of factors influencing delayed childbearing, offering a clear picture of the multifaceted decision-making process. By synthesizing historical context, societal dynamics, psychological intricacies, and policy implications, we have underscored the multidimensional nature of this phenomenon. Our findings emphasize that delayed childbearing reflects evolving aspirations, changing expectations, and expanding opportunities in the modern world. Recognizing the long-term consequences and major factors in this trend is crucial. Proactive measures are essential to navigate the challenges it poses and harness its potential benefits. This includes the development of supportive policies, understanding the emotional nuances involved, and fostering open dialogues among individuals and within society as a whole. By doing so, we can create an environment that not only embraces the diverse pathways to parenthood but also thrives in the presence of delayed childbearing. In the ever-evolving landscape of parenthood, research, understanding, and empathy will be the cornerstones of a society that not only adapts to the complexities of delayed childbearing but also flourishes in its presence.
